# Lightweight and Efficient Dynamic Cluster Head Election Routing Protocol for Wireless Sensor Networks

**DOI:** 10.3390/s21155206

**Published:** 2021-07-31

**Authors:** Mudathir F. S. Yagoub, Othman O. Khalifa, Abdelzahir Abdelmaboud, Valery Korotaev, Sergei A. Kozlov, Joel J. P. C. Rodrigues

**Affiliations:** 1 Faculty of Computer Science & Information Technology, University of Omdurman Islamic, Khartoum 825109, Sudan; mudathir.f.yagoub@sustech.edu; 2 College of Graduate Studies, Department of Computer Science, Sudan University of Science and Technology (SUST), Khartoum 825109, Sudan; 3 Department of Electrical and Computer Engineering, International Islamic University, Gombak 53100, Malaysia; khalifa@iium.edu.my; 4 Department of Information Systems, King Khalid University, Mayahel Aseer, Abha 62529, Saudi Arabia; aelnour@kku.edu.sa; 5 ITMO University, 197101 Saint Petersburg, Russia; korotaev_v_v@mail.ru (V.K.); kozlov@mail.ifmo.ru (S.A.K.); 6 Federal University of Piauí (UFPI), Teresina 64049-550, PI, Brazil; 7 Instituto de Telecomunicações, 6201-001 Covilhã, Portugal

**Keywords:** wireless sensor network, cluster head election method, energy consumption, service redundancy phenomenon, dynamic self-organization method

## Abstract

Wireless Sensor Networks (WSNs) have gained great significance from researchers and industry due to their wide applications. Energy and resource conservation challenges are facing the WSNs. Nevertheless, clustering techniques offer many solutions to address the WSN issues, such as energy efficiency, service redundancy, routing delay, scalability, and making WSNs more efficient. Unfortunately, the WSNs are still immature, and suffering in several aspects. This paper aims to solve some of the downsides in existing routing protocols for WSNs; a Lightweight and Efficient Dynamic Cluster Head Election routing protocol (LEDCHE-WSN) is proposed. The proposed routing algorithm comprises two integrated methods, electing the optimum cluster head, and organizing the re-clustering process dynamically. Furthermore, the proposed protocol improves on others present in the literature by combining the random and periodic electing method in the same round, and the random method starts first at the beginning of each round/cycle. Moreover, both random and periodic electing methods are preceded by checking the remaining power to skip the dead nodes and continue in the same way periodically with the rest of the nodes in the round. Additionally, the proposed protocol is distinguished by deleting dead nodes from the network topology list during the re-clustering process to address the black holes and routing delay problems. Finally, the proposed algorithm’s mathematical modeling and analysis are introduced. The experimental results reveal the proposed protocol outperforms the LEACH protocol by approximately 32% and the FBCFP protocol by 8%, in terms of power consumption and network lifetime. In terms of Mean Package Delay, LEDCHE-WSN improves the LEACH protocol by 42% and the FBCFP protocol by 15%, and regarding Loss Ratio, it improves the LEACH protocol by approximately 46% and FBCFP protocol by 25%.

## 1. Introduction

In Wireless Sensor Network (WSN) environments, small, energy-efficient, low-cost sensor nodes are exchanging the sensed data with the base station (BS) effectively [1,2]. Nowadays, WSNs are considered a new technology issue when machines, humans, and the environment are independently integrated. Consequently, the evolution in memory, processors and microelectronics devices enables the performance of different programmed tasks, in turn allowing sensing and computing elements to be integrated into small devices [3]. Moreover, separated sensors formed WSNs [4,5] for environmental monitoring or physical conditions, such as temperature, pressure, precision agriculture, sound, machine surveillance, intelligent buildings, facility management, habitat monitoring, preventive maintenance, logistics, and transport, etc. Subsequently, their data bypass via the network to the main location cooperatively [6]. Battlefield supervision as an example of military applications encouraging the development of wireless sensor networks. Today, these networks are used in many manufacturing and consumer applications, such as control and process monitoring, healthcare, and medicines [7,8,9], etc. However, energy, distance, and time delay are considered the main challenges of WSN. Furthermore, in most WSN cases, a non-rechargeable battery is used as a sensor power resource. Thus, distances among nodes are highly increasing energy consumption. Accordingly, the cluster-head approach as the core of typical clustering algorithms is used to reduce distances among nodes, decrease service redundancy phenomena, and achieve a balanced distribution for energy consumption. Wrong selection to the cluster head often causes early depletion of the network energy [10]. Additionally, the attackers can gain full control of whole the network, without any need to overtake other nodes, when they gain control of all cluster heads. Conversely, proper cluster head electing may decrease the energy consumption, ensure a constant data flow, increase the data integrity level, and prolong the network lifetime [10]. As is well known, as the distance among nodes and base stations increases, the time delay is increased. Likewise, the CH must be selected in a way that is spatially close enough to the base station as well as the sensor nodes. As a result, the time delay can be reduced effectively, and the speed of the data transmission can be increased accordingly [1]. The excessive calculations for the measuring methods, which are used to ensure the CH election accuracy, and applied by most of the previous studies —especially in stationary networks— such as distance among nodes and base station, signal strength, hop count, node density, have been deliberately ignored. However, the drawbacks of those protocols are the focus on the degree of accuracy in choosing the cluster head by performing complex mathematical operations and measuring multiple factors, causing delays, and draining of the network energy that is supposed to be provided. On the other hand, the LEDCHE-WSN algorithm checks the remaining energy just to ensure that a CH candidate is alive, without adding a burden of calculation and comparison for the remaining energy of the CH candidate with all the other sensor nodes. Instead, good architecture design (known as topology control, see Section 4.1) makes the connectivity more simple and flexible. Furthermore, our proposed architecture is enhanced by using a single-hop model at the cluster core, where it has a small size and a short distance among nodes. Moreover, using a multiple-hop model at the cluster core (as it is in the LEACH protocol) causes multi congestion points in the cluster, which in turn result in quick depletion of the nodes’ energy. Therefore, the multi-hop clustering model is used in the cluster edge (between the SN and BS) to achieve good scalability, which the single-hop clustering model does not achieve. In addition, a combination of three different methods (random, periodic, and checking the remaining power at each) are used to make the routing protocol lightweight and more efficient, as detailed in different parts of the manuscript. In a summary, the proposed model is distinguished by simplicity, ease, and lightness, and these are achieved by avoiding the complexity burdens resulting from intense calculations.

From previous studies, it may be concluded that WSNs are still immature in several aspects. WSNs still suffer from power consumption as a major problem. In the same way, clustering is a common approach used to reduce delays, loss ratio, and power consumption. However, it lacks a method for electing an optimum cluster head to enhance the data aggregation efficiency. Furthermore, inefficient cluster head election leads to the high-power consumption of the sensor nodes. Besides, the extensive calculation in the cluster head election process, such as node centrality measure, hop count, and density, results in energy depletion quickly, consequently shortening the lifetime of the network. Finally, the high delays in the WSNs are occurred due to incorrect energy-based path selection.

To solve these downsides in existing routing protocols for WSNs, the proposed protocol LEDCHE-WSN constructs the coming objectives. It introduces a simple, light, and easy deploy algorithm. It reduces power consumption by using the clustering approach with efficient cluster head election in WSNs and, therefore, the lifetime of the WSNs is prolonged. Additionally, the LEDCHE-WSN protocol finds an optimal method to send a data packet to the sink node with minimum delay and routing overhead. It also enhances security during the CH election process. In this sense, this work aims to reduce power consumption, enhance network delay, and strengthen the security side. Therefore, the main contributions of this study are the following:

The proposal of LEDCHE-WSN routing protocol introduces a new random method to elect the CH candidate by checking its remaining power at the first round, to skip dead nodes and to complexes tracking the CHs by attackers, unlike a common random method, which elects the CH randomly and blindly (without checking);

The proposal of a protocol using a combination of a random and periodic method per round to gain their advantages during the CH election process. Moreover, both random and periodic electing methods are preceded by checking the remaining power to skip dead nodes, and continue in the same way periodically with the rest of the nodes in a round/cycle to distribute the power load equally among network sensors.

The proposed method is distinguished by deleting the failure and/or dead nodes from the network topology during the re-clustering process, to address the black holes and routing delay problems. In turn, it results in a waste of time and a waste of energy, due to additional control messages in repetitive checking in each round/cycle for useless nodes if they are not deleted from the topology list. The mathematical modeling and analysis of the proposed algorithm is introduced to illustrate and strengthen the proposed approach. Performance assessment of the benchmark protocols and used in evaluating and validating the proposed LEDCHE-WSN routing protocol.

The rest of the paper is organized as follows. Some background about the mechanisms required, and clustering approaches in WSNs, are described in Section 2. Section 3 presents the related work about CH selection protocols for WSNs under different categories. Section 4 introduces the proposed network model and its architectural characteristics. The proposed LEDCHE-WSN protocol is presented in Section 5. Performance evaluation and results analysis are presented in Section 6. Finally, Section 7 concludes the paper and suggests future research works.

## 2. Concepts of Clustering Approaches

**Figure 1:**
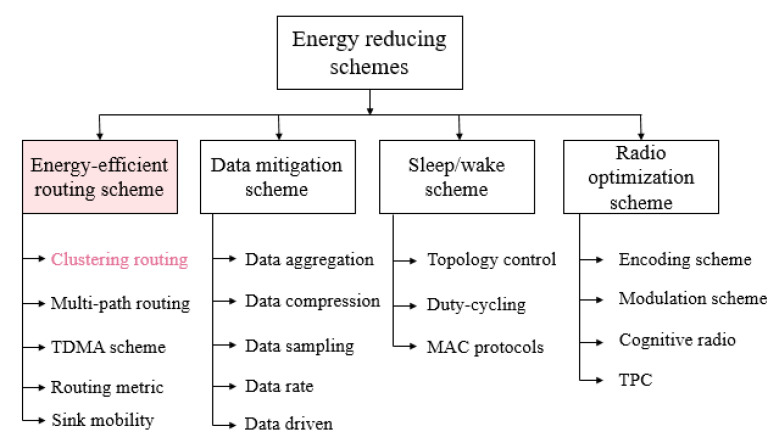
Classification of energy reducing schemes in WSNs.

The clustering process works to divide the sensor network into many groups. Each group elects its cluster head, which is considered as a leader to represent the cluster in the process of sending data. Cluster head election can be set by the network designer or by sensor nodes. Clustering techniques could be used to solve many constraints in wireless sensor networks by authorizing the sensor’s architecture differently. It is performed by aggregating the sensors into clusters, and giving a specific task to each sensor, then transferring data to upper levels. The main goals of clustering techniques are to gain high energy efficiency, reuse bandwidth, target tracking, data collection, and guarantee a long lifespan of the network. Furthermore, clustering is considered as one of the common techniques used to face the power consumption and redundancy phenomenon caused during the service access and transmission process. Thus, optimized clustering reduces redundancy within the network and saves energy significantly [11]. Finally, clustering is the key routing technology to economize energy consumption [12]. Figure 1 illustrates the basic and common schemes used to reduce energy consumption, including the cluster-based architecture under study.

### 2.1. Cluster Head and Clustering Operations

Clustering systems divide sensor networks into many groups. Each group elects a specific node as a cluster head to represent the group in the data sending process, and the remaining nodes are regular nodes [1].

The rule in operations of clustering considers several rounds. Every round comprises two stages; the preparation stage and the steady-state stage. The first stage involves a mass formation and a cluster head (CH) election process, and the second stage performs the process of data transmission. The CHs’ energy consumption generally is high, due to the data collection and transmission process [13]. As the consumption of CHs is high, therefore it is, necessary to rotate this functionality.

### 2.2. Formation of a Cluster

Cluster formation considers two stages, a cluster head election process, and nodes identification to the elected cluster heads. A cluster head organizes all the transmissions within the cluster. Thus, a cluster head deals with delivering the collected packets for the cluster, i.e., inter-cluster traffic [1].

### 2.3. Homogeneous and Heterogeneous Sensor Networks

WSN is called homogeneous if all sensor properties are matched. Sometimes, different types of sensors may coexist in the same network. Heterogeneous WSNs (HWSNs) include sensor nodes with different characteristics, whereas all the devices in homogeneous WSNs have the same connectivity and computing capabilities. HWSNs allow a variety of operating environments, so they are useful for many applications [8].

### 2.4. Single-Hop vs. Multi-Hop Models

**Figure 2:**
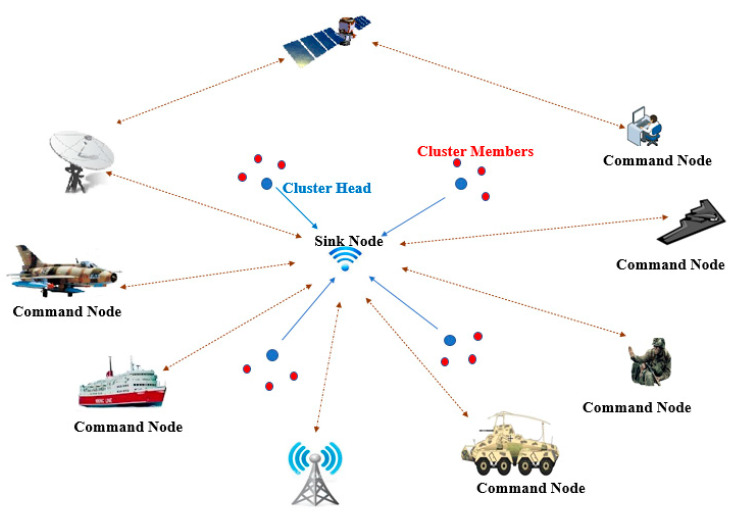
Military architecture for monitoring application using the single-hop clustering model in WSNs.

Data are transmitted between different nodes and the sink node in one of two methods, the single-hop model and the multi-hop model. In the single-hop model, all sensor nodes transmit their data to the sink node. This model speeds up the data process and makes the implementation easy, resulting in reduced energy consumption. However, single-hop models are infeasible in large-scale environments, as the transmission cost consumes high energy levels, and in the worst case the sink node cannot be reached. Figure 2 illustrates an example of a military surveillance application using the single-hop clustering model.

**Figure 3:**
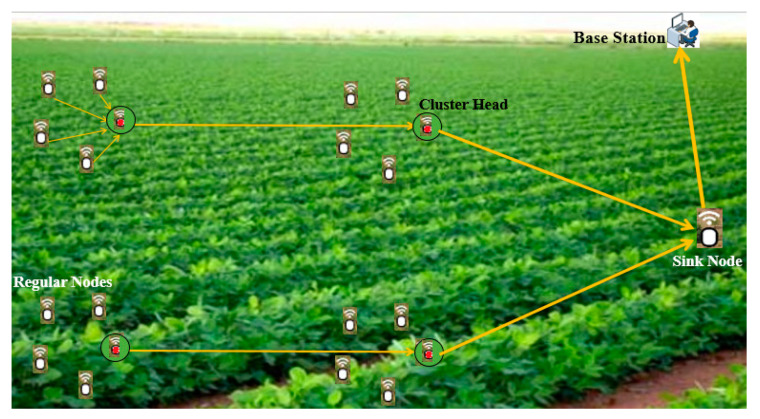
Precision agriculture architecture for monitoring application using the multi-hop clustering model in WSNs.

On the other side, the multi-hop clustering model keeps the sensor nodes at a low level of energy consumption, as multiple cluster heads collect data and transfer them to the sink node. However, the synchronization of multi-hop time in the cluster core produces a very high load and data transmission among nodes, causing more packet delay and packet loss and, therefore, quick energy depletion. Consequently, the multi-hop clustering model is suitable for a large-scale area network [14]. Figure 3 shows an example of a precision agriculture application using the multi-hop clustering model.

## 3. Related Work

Currently, great attention has been gained by WSNs in wide applications and research areas, such as industrial, environmental monitoring, and healthcare applications. Due to the limited sensors, communication capabilities between them must avoid the exhaustion of battery energy. To the best of the author’s knowledge, the LEACH protocol [15] is the first protocol based on a clustering approach, which uses random selection of cluster heads to distribute the power load equally among network sensors [16]. It also localizes coordination to make the dynamic networks much more scalable and robust, and the routing protocol includes a data merging process to reduce the information amount sent to base stations. The results show that LEACH can reduce energy more than eight times as efficiently in comparison with conventional routing protocols. In addition, LEACH can distribute power dissipation evenly across the sensors. However, in the optimum results [17], note the distribution of LEACH’s cluster heads is uneven and, in some areas, the cluster heads are very concentrated. There are some areas where there is no distribution of cluster heads. Moreover, some nodes are too far from the cluster heads, which leads to wasting energy in data transmission. In the same way, the FBCFP protocol uses neuro-fuzzy rule-based clustering concepts to perform cluster-based routing [18]. Energy modeling is utilized to perform cluster formation, in WSNs, for efficiently routing packets through the application of machine learning using a convolutional neural network with fuzzy rules for weight adjustment and, hence, the network lifetime is prolonged. However, the focus on the degree of accuracy for choosing the cluster head by performing complex mathematical operations and measuring multiple factors results in delays and drains the network energy that is supposed to be provided. Similarly, DFLER [19] uses fuzzy logic and the ECATCH algorithm to reduce the workload of the cluster head and produce a reliable, accurate results. However, sending all sensor data to a specific CH leads to a bottleneck problem. One strength of NCCM-DC [20] is producing a high-performance protocol to enhance energy efficiency and achieve transmission reliability, but a very high load is produced from the synchronization of multi-hop time. Additionally, FEMCHRP [21] elected the cluster heads using the fuzzy logic and Dijkstra algorithm to perform the data transfer process by selecting the shortest energy path. However, repetitive use of two methods in the CH election process, at each round, leads to significant energy drain. To enhance the total network lifetime and improve the LEACH protocol, a new algorithm was proposed, based on the node rank (NR) algorithm, called NR-LEACH [5]. However, the repetitive calculation for the path cost, and the number of links among the nodes in each round, depleted the NR-LEACH nodes’ energy quickly during the CH election process. Another promising approach from Firefly with Cyclic Randomization (FCR) algorithms is proposed in [1], to enhance an effective CH election method considering energy, delay, and distance in WSNs. However, the random method used in cluster head election itself results in great delays due to the black hole problems, and the extended amount of time needed to get out from those holes. It is important to highlight that the work in [22] exploits the transmission range for the control messages and the average number of clusters to prolong the network lifetime effectively. However, repetitive calculations of the transmission range of the control messages and an average number of clusters in each round, causing quick depletion of the network’s power, and greatly delaying the operations. Similarly, RTCP [23] exploits the redundant sensors in the same area and clusters them. To keep the backbone connected, the redundant nodes are stopped, and some active nodes are maintained. However, the application selected the Tcl value, which affects the number of ready-made groups. When the Tcl is small, few groups are made up. Thus, finding the true Tcl value shows the minimum number of groups, so, when the nodes do not have the same redundancy ratio, it became difficult. On the other hand, DFLCHES [24] introduces an effective CH election that enhanced the network lifetime by exploiting fuzzy clustering and the Genetic Algorithm (GA). However, the extensive calculation for measurement parameters, such as distance, hop count, centrality measure, and node density, depleted the energy quickly and shortened the network lifetime. Likewise, for performing efficient routing in IoT, a new Cluster Formation based on Neuro-Fuzzy Rule is introduced in [18]. The proposed model is characterized by simplicity, ease, and lightness and these are achieved by avoiding the complexity burdens resulting from intense calculations that used to ensure the accuracy of the cluster head election process, such as node density, hop count, centrality measure, and messaging, which causes delays and drains the network energy that is supposed to be provided. Experimental results reveal the proposed routing algorithm performs better in terms of packet delivery ratio, energy utilization, network lifetime, and delay. However, many proposed algorithms are using different factors and strategies for selecting cluster heads, and their role is rotating through sensor nodes to attain load balancing, resulting in prolonging the network lifetime. Based on the factors, cluster head election strategies are commonly classified as adaptive, deterministic, or combined. The related works’ summary for the clustering protocols is shown in Table 1.

It may be seen in Table 1 that most models that adopted clustering technology to face power consumption and service redundancy issues; the power saving results were high. The dynamic cluster head election technology is one of the most0 effective methods to achieve energy-saving and reduce service redundancy problems. Most of the latest technologies that enhanced LEACH protocol adopted the measuring method, such as measuring the signal strength, residual energy, node density, hop count, the distance between nodes, and base station. However, the drawbacks of those protocols are the focus on the degree of accuracy in choosing the cluster head by performing complex mathematical operations and measuring multiple factors, which increases the complexity. All previous approaches include the expense of adding more burdens on the network, which quickly the energy that is supposed to be provided. The protocols that adopted the random method in a cluster head election process caused much more delay, due to the false election of the failure nodes (black holes) and much more time needed to get out of those holes. Conversely, protocols that elect the cluster head periodically, distributing energy among network nodes fairly, extend a node’s life and, in consequence, the network lifetime. On the other hand, this weakens security when the attacker gains control of all the cluster heads, as they will gain full control of the network without having to overcome any other nodes [10]. A few of the reported protocols have adopted combined metrics, which mix architectural and algorithm metric solutions. The combined metric is a good approach that overcomes the limitations of periodic, random, and measuring approaches, while at the same time gaining their advantages [15].

## 4. Proposed Network Model

**Figure 4:**
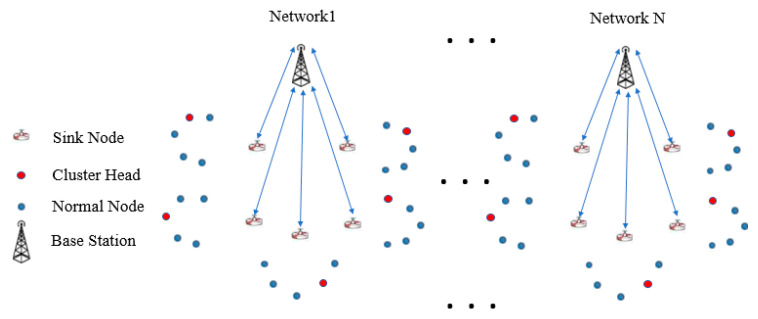
Illustration of the LEDCHE-WSN architecture.

The used network to evaluate the proposed clustering protocol is composed of *n* nodes uniformly distributed in a field Q × Q square meters. The existence of a link is specified only by the distance among nodes, without taking into account disruptions due to obstructions and interference of wireless signals. A cluster is formed by specifying the cluster head and its cluster members. The cluster member is directly connected to the cluster head. Figure 4 illustrates the proposed network architecture.

### 4.1. Nodes Placement

Nodes deployment pattern makes a significant positive impact on the performance of a wireless sensor network. A sensor node is deployed either by placing it in a precise location depending on the application scenario, or by deploying it randomly. A sensor node is also positioned based on the monitored data. For example, in a remote agriculture application, a sensor node is placed manually in the field. If node placement changes continuously, a routing protocol will suffer from the frequent creation and broken of many links. Moreover, if nodes are deployed densely, a single node has many neighbors that can be directly connected, which will consume more power energy and add more burden to the Media Access Control (MAC) protocol due to the repetitive sending to a large-range.

To overcome the above issues, topology control is applied. The idea behind using topology control is to control a set of neighbor nodes. Sometimes, a topology is controlled by placing a set of neighbor nodes manually. Topology can also be controlled by creating hierarchies in a network or clustering, by controlling transmission power. A topology is considered based on the used communication technology and the system network requirements. In some scenarios, a hybrid topology is the best option.

The proposed protocol is a hybrid topology, and a combination of centralized and distributed approaches is adopted. Moreover, the topology is controlled by placing a set of neighbor nodes manually. The proposed network model is discussed in more detail in Section 4.2, Section 4.3, and Section 6.2.

### 4.2. Network Model Assumptions

Regarding the proposed protocol, some basic reasonable assumptions are adopted to define the scope of the proposed network model as follows:

- The network has *N* sensors distributed in the Q × Q square field;
- Each node has a unique ID;
- The sink node (SN) location is known by all the sensors, and vice versa;
- The network nodes are stationary;
- All the network nodes are homogeneous and energy-constrained;
- The SN of each cluster is located at the center of the cluster, so all nodes of the cluster can be connected with it using the single-hop model method;
- The SN has unlimited energy resource;
- The BSs are located at the center of each network. The BSs collect their data from rechargeable SNs;
- One BS is used for each 100 × 100 m network size;
- Regular sensors can communicate directly with their cluster head (CH);
- The size of a cluster in a network concerning the number of regular sensors is equal;
- In the beginning, all sensor nodes have the same energy level (maximum) and each of them can become the CH or a regular node;
- All the nodes have the capability with appropriate distance to send the information to the sink node.

### 4.3. Network Architecture Designing

The network architecture is designed as depicted in the scenario shown in Figure 4, and it is configured according to the assumptions mentioned above (Section 4.2). The proposed protocol is implemented in the sink node gateway to select an optimum cluster head dynamically. Moreover, the location of the nodes, including the SN, is configured when the network is initialized and the network nodes are stationary. Therefore, when the SN elects the optimum CH, it informs all nodes about the location of the current CH. However, to scale the proposed network, only the new cluster is added (with its corresponding SN) in the specified direction to the nearest cluster edge, and the first configuration is performed manually. Furthermore, the network topology list for each cluster in the SN is updated dynamically when dead nodes are deleted during the dynamic re-clustering process. The uniform deployment was essentially adopted to locate the SNs at the center of each cluster, to enable all the regular nodes to communicate to it directly and, in the same way, the BS was located in the center of the SNs for the same reason. Additionally, the area of each cluster is in an appropriate size to enable all regular nodes to communicate with one another directly and effectively. Furthermore, the adopted periodic election policy keeping the view of uniform deployment of nodes until close to the end of the network’s life. The proposed algorithm in Section 5 will explain further how to implement the proposed network.

#### Proposed Lightness Architecture and Protocol Efficiency

The philosophy for choosing an appropriate network architecture and its data transmission model has a major impact on the network lightness and efficiency of the proposed protocol. According to the recommendations of a survey conducted in [32], it becomes clear that the lightest architecture among different architectures is the hybrid architecture, as it acquires the advantages of the distributed and centralized approaches while exceeding their particular limitations. Therefore, the researchers worked to reduce the number of nodes in each cluster to overcome the issue of the energy bottleneck region around the sink node. Similarly, the acceptable number of sink nodes is increased to obtain the advantages of the distributed approach. On the other hand, when the number of clusters is small, intra-cluster distance is becoming too far and it will cause extra power consumption for data transmission and data loss. In addition, an overlarge number of clusters leads to redundancy when several cluster heads send the same data to the base station (BS) [33]. Experiments should be performed to determine the optimal number of sink nodes and the optimal number of regular sensors in each cluster according to the energy consumption of the whole network lifetime.

A sink node of each cluster is located at the center of the cluster to aggregate data from all the nodes effectively. Furthermore, to make the network lightweight and less power consuming, to improve the data transmission process, and to adjusting the deployment to be easy, a hybrid-clustering model is adopted. A single-hop clustering model is used in the cluster core (between all the sensor nodes and the cluster head). However, its drawback appears when the network size grows in ways such as consuming much more power and increasing the dropping ratio due to the large distances among nodes. Thus, a single-hop method is adopted at the core of the cluster, where nodes are a small size and have a short distance between one another. Conversely, to achieve connectivity and scalability, which the single-hop clustering model does not achieve, the multi-hop clustering model is used in the cluster edge (between the CH and BS via the SN). The reason for adopting a multi-hop clustering model in the cluster edge comes from the fact that synchronization of multi-hop time in the cluster core produces a very high load and data transmission among nodes, causing more packet delay and packet loss, and therefore, quick energy depletion.

In the same way, to make a network more efficient, the proposed CH election algorithm has avoided the mathematical complexity and many factors considered for precision in electing the optimal CH. They are commonly used, causing much more power consumption and delays. Therefore, the periodic method has been used for electing the cluster head, assuring the node is alive by checking the remaining power without making any accounting or comparing load with other nodes. Due to all these procedures, the network lightness and efficiency of the proposed protocol are guaranteed.

## 5. Proposed Dynamic Cluster Head Election Algorithm

The philosophy of the proposed model is based on improvement in two different directions, the first is designing an appropriate architecture that helps in the lightness of the network and its ease of expansion, which is discussed, in detail, in Section 4.3. The second is the enhancement in the algorithm that operates on that architecture, which will be discussed in this section.

The proposed dynamic clustering head election algorithm is structured from two methods. The first is introduced to elect the optimum cluster head, while the second method is introduced to make dynamic self-organization for the clusters when the CH candidate dies or fails. Subsequently, the energy level will be distributed equally among the nodes, the power consumption will be reduced, and, therefore, the lifetime of the network will be improved.

### 5.1. Proposed Optimum Cluster Head Election Method

There is a set of well-known techniques used to select the appropriate cluster head (CH) of each cluster. To explain how the proposed algorithm works it will focus on a single cluster and then generalize the idea to multiple clusters. The pseudo-code presented in Algorithm 1 explains how the proposed method is working in a single cluster.

After the network architecture is designed and its configuration is completed according to the assumptions (Section 4.3), the proposed protocol is implemented in the sink node gateway to select an optimum cluster head dynamically.

The basic idea in the proposed method is to distribute the energy approximately equally among the cluster nodes in each round/cycle, along with the complexity of tracking the CHs by the attackers.

**Figure 5:**
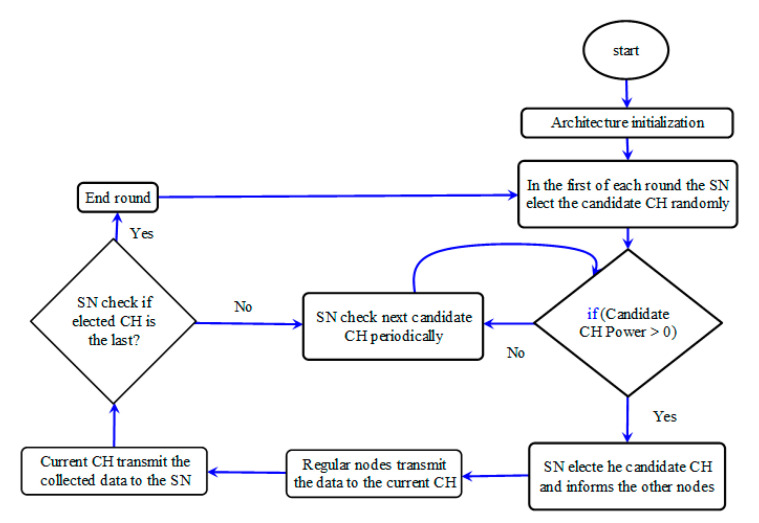
The proposed Optimum Cluster Head Election method’s data flow diagram.

At the beginning of each round/cycle, the corresponding SN elects the CH candidate randomly (just to determine the CH candidate priority; the election is not finished yet), then the CH candidate power is checked. If the CH candidate > zero, that meaning the CH is alive, the SN elects it as a current CH and informs the other nodes. Otherwise means the CH candidate is dead, therefore the CH candidate is skipped and the next node is checked periodically until the end round is reached, re-organizing the cluster after deleting dead nodes from the topology list as illustrated in Section 5.2. Accordingly, the regular nodes transmit their data/service to the current CH and, in turn, the current CH transmits the collected data/service to the corresponding SN. After the CH is elected, the SN makes another check to ensure whether the current CH is the last node in this round/cycle or not; that happens when the next node (during the periodic checking) is itself the CH candidate that was elected randomly at beginning of the first round. If the current CH is not the last one in the round, the next CH candidate is checked periodically. Otherwise, it means the first round is finished, and the SN elects the CH candidate for the next round randomly, etc. Figure 5 shows the proposed Optimum Cluster Head Election method’s data flow diagram, which illustrates all the idea mentioned above, and how this method works.

Testing the residual energy of the CH candidate with a specific threshold is one of the common solutions. However, the process of testing these near-death nodes is a waste of time and energy, due to the additional control messages in repetitive testing in each round/cycle for useless nodes if they are not deleted from the topology list, which opens the door for further research to address these challenges effectively. Therefore, this issue is considered as a future work, as mentioned in Section 7.

Note that the proposed Optimum Cluster Head Election Method lifts from the regular nodes the processes of selecting the next CH node, and the process of notifying the rest of the cluster nodes about the elected CH. Instead, all previous functions were assigned to the rechargeable SN nodes. Contrary to most of the previous studies where the cluster nodes participate in selecting the next CH node and then notify the rest of the cluster nodes to fulfill these roles in these models, cluster nodes keep all the information of the topology list, which leads to a significant and effective drain on cluster nodes’ energy.

In short, the main new ideas of the proposed method can be summarized as follows:

(i) Introduce a new random method to elect a CH candidate; the new method checks the remaining energy for the CH candidate (not the CH itself) which is elected randomly (at the beginning of each round/cycle) to overcome the problems of the black holes. This is contrary to the common random method, which elects the CH randomly and blindly. (ii) Furthermore, the idea behind using the random election method at the beginning of each round is to strengthen the security side and complexity of tracking the CHs by the attackers. Moreover, the random election method is used only at the beginning of each round, and not over the length of the round time. (iii) The next new idea is that, after starting the beginning of the round by using the random method, the rest of the nodes are electing periodically, also checking the remaining energy to skip the dead/failed nodes and save energy and time. Moreover, using the periodic method during the rounds/cycles distribute the energy load equally among network sensors.

#### Benefits of Random and Periodic Methods Combination

After network creation is completed, the sink node elects the CH candidate randomly at the beginning of each round, to complicate the tracking of the CHs by attackers, who seek full control of the network and all the data collected in the CHs without the need to obtain any other nodes. Moreover, and after the first CH is elected by the SN, the rest of the CHs are elected periodically until the end of the first round, to achieve a good load balance for the network, which prolongs the node life and extends the network lifetime. Then, the CH is randomly elected at the beginning of the second round, and the process continues in the rest of the rounds. Thus this combined metric is a good way to overcome the limitation of each one, and at the same time maintain their strengths.

The benefits of mixing the previous two methods (random and periodic) can be summarized as follows:

- Strengthening the security side by electing the CH candidate randomly at the beginning of each round, which complicates the attackers’ tasks and attempts as tracking CHs to obtain information;
- Distributing energy among the network nodes fairly, using a periodical election method during each round extending the network lifetime;
- The simplicity, ease, and lightness of the proposed model avoids the complexity burden due to more mathematical operations and messaging for accuracy, which drains the network energy that is supposed to be provided.

**Algorithm 1** Working of the proposed method in a single cluster**Input**: *ClusterNumber, DevicePower, DeviceId, SIZEOFCLUSTERS* **Output**: *Assigning the optimum Cluster Head* fn_AssignClusterHead (ClusterNumber, DevicePower) //Function assign Cluster Heads randomly at the beginning of each cycle and periodically with the //rest BEGIN    R = Generate a random number from [SIZEOFCLUSTERS]    FOR each sensor i elected randomly R at the beginning of each cycle DO   DeviceId = ClusterElements [ClusterNumber][i]   ClusterHeadPower = DevicePower [DeviceId-1] ->dRemainingPower       IF the **candidate** CH is a live THEN          ClusterHeadID = DeviceId            Else        CALL fn_DeleteDeadSensors (ClusterNumber, DevicePower)             END IF   RETURN ClusterHeadID    END FOR END


To calculate the energy consumption of the whole network and the formula of its mathematical modeling, it is necessary to calculate the energy consumed for the two sensor points and the formula of its mathematical equation, then expand the equation to include the whole network. The environmental results sensed by the two sensor points should be sent to the central monitoring unit called the sink node (SN). The energy needed to transmit the data between two sensor points $E_{nerg}2sens$ can be expressed as:

(1) $$E_{nerg}2sens=E_{trans}\cdot N_{trans}\_dbits+\varepsilon amplifcoe\cdot dis^{\alpha }$$

where $E_{trans}\left(J/b\right)$ is the wasted energy in transceiver operation, $N_{trans}\_dbits$, is the number of transmitted data bits, $\varepsilon amplifcoe\left(J/b/m^{2}\right)$ is an amplification coefficient used to ensure the lowest bit error rate due to achieving the reliability of receiver, α is a factor of 2 to 5, depending on the transmission environment of the networks, and *dis* refers to the distance between two sensor nodes, which can be expressed as:

(2) $$E_{trsans}=Volt_{wrk}\cdot curr_{trans}/T_{data\text{-}rate}$$

where $V_{w}$ indicates to working voltage, curr indicates to current for transmission, and $T_{data\text{-}rate}$ indicates to the transmission data rate.

In contrast, we can express the energy consumed to receive the data from the other side $E_{reciv\text{-}data}$ as:

(3) $$E_{reciv\text{-}data}=E_{trans}\cdot N_{trans}\_dbits$$

Equation (1) reveals that the consumed energy in the fixed distance is directly proportional to the data bit numbers. Conversely, the greater distance between the two nodes means more energy has been consumed.

**Schemes of Sensing**

The wireless sensor concept means the possibility of physical sensing, and the ability to process the data obtained and communicate the results wirelessly. In recent years, many power-saving schemes have been introduced which assume that data acquisition and processing consume far less power than communication [34]. All network sensors are constrained, and their components consume a specific energy amount, taking into account the increasing growth network. Therefore, the process of supplying power and ensuring the proper operation of WSN becomes very difficult. Hence, building structures of effective wireless networks for energy efficiency networks is critical.

By the network scheme switching, power is saved substantially at the network level. Two different network schemes are reviewed as follows:

**Scheme 1 (Single Cluster Algorithm):**

The data points obtained from all sensor nodes are transmitted to the CH, and $E_{allSens\text{-}CH}$ energy consumption can be calculated, in this case, as:

(4) $$E_{allSens\text{-}CH}=\sum_{n=1}^{N}[(E_{trans}+\varepsilon amplifcoe\cdot dist_{n\text{-}sn}^{{}^{\alpha }})\cdot N_{trans}\_dbits]$$

where N indicates the number of nodes, $dist_{n\text{-}sn}$ indicates the distance between nodes and the sink node, and $N_{trans}\_dbits$ indicates the obtained data bits.

For example, Figure 4 illustrates the Single Cluster Algorithm. Consider it as a process of managing and monitoring the precision agriculture platform, where sensors obtain humidity and temperature. In each platform, the sensed humidity and temperature are directly transmitted to the communications management unit SN without relay and routing.

### 5.2. Proposed Dynamic Re-Clustering and Self-Organization Method

**Figure 6:**
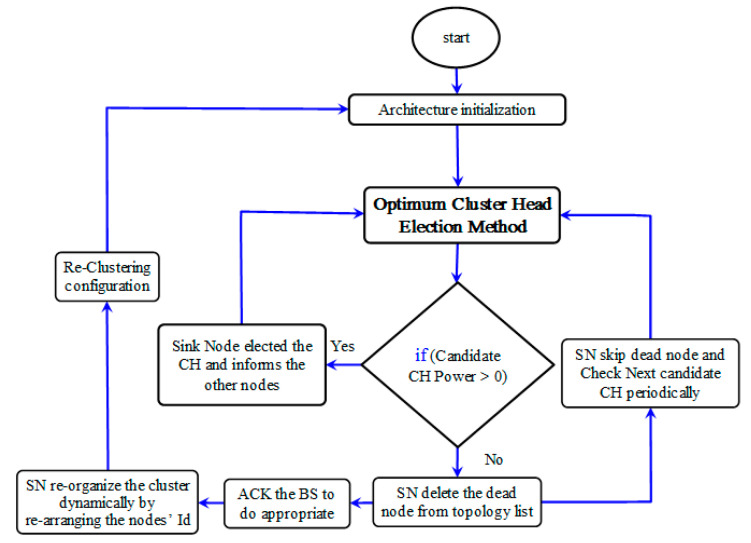
The proposed Dynamic Cluster Self-Organization Method’s data flow diagram.

During the process of the proposed Optimum Cluster Head Election Method, and when the SN checks the CH candidate, a result greater than zero means it is alive and, hence, the SN elects it as a current CH and informs the other nodes. Otherwise means the CH candidate is dead or failed. Thus, the SN deletes the CH candidate from the topology list, sends a report/feedback to the Base Station (BS) to do the appropriate, and re-organizes the cluster dynamically by re-arranging the node’s ID accordingly. Furthermore, any nodes that cannot transmit their status are considered as dead/failed nodes, so they will also be skipped and deleted from the topology list; the checking of the rest of the nodes will continue periodically. In short, the new idea of the proposed method is that, when the CH candidate is dead/failed, the proposed protocol skips the node and deletes it from the topology list to address the black holes and routing delay problems which cause the waste of time and energy, due to additional control messages for useless nodes which are not deleted from the topology list. Next, the proposed method reorders the nodes and sends feedback to replace the node or do the appropriate. Figure 6 shows the proposed Dynamic Cluster Self-Organization Method’s data flow diagram.

#### 5.2.1. Mitigation of Energy Consumption during Cluster Reconfiguration

Occasionally, the CH consumes more energy due to the cluster preparation stage and many data transfers. Re-clustering is proposed to distribute the CH role among the sensor nodes equally and extend the network lifetime. However, re-clustering increases power consumption, due to additional control messages, and delays real-time data transmission [35,36]. The proposed model provides a solution that involves deleting dead or failed nodes from clusters, which saves wasted energy in sending messages to a dead or failed node. It also saves wasted time to get out of the false election of the failed nodes (black holes). As a consequence, it will not consider dead or failed nodes in the upcoming cluster reconfiguration process.

#### 5.2.2. Dead Nodes and Re-Clustering Organization

During the verification of the remaining energy of each node, after discovering a dead node the proposed algorithm deletes the dead node from the topology list and then notifies the Base Station. Therefore, the dead node will not be checked the next time, thus overcoming the false election problem (black holes), which wastes energy and time. After this deletion, the cluster is reconfigured and organized according to the new situation, which makes the cluster lighter and more energy-saving. The pseudo-code presented in Algorithm 2 shows how to delete the dead nodes from the topology list, and the processes of Base Station notification and re-clustering configuration.
**Algorithm 2** Deletes the Dead Sensors from the Cluster**Input**: *ClusterNumber, DevicePower, SIZEOFCLUSTERS* **Output**: *Delete the Dead Sensors from the Cluster* fn_DeleteDeadSensors (ClusterNumber, DevicePower) // Function delete the Dead Sensors from the Cluster and sends a report to the Base Station (BS) BEGIN     FOR each sensor i starting from the dead sensor DO      Delete the Dead Sensors by Copy next element value to current element      ClusterElements [ClusterNumber][i] = ClusterElements [ClusterNumber] [i + 1]    END FOR    Decrement SIZEOFCLUSTERS by 1    ClusterElements [ClusterNumber] [SIZEOFCLUSTERS--]    Send a report telling the Base Station that the DeviceId (in the ClusterNumber) is dead END

#### 5.2.3. Multiple Cluster Organization

The previous operations were concerning a single cluster, whereas in the case of multiple clusters, the cluster is determined to elect the appropriate CH first, then move among the rest of the clusters to apply the same scenario. Moreover, data of all the clusters are sent to the sink node. The pseudo-code of Algorithm 3 presents how to move between the other Ccusters to elect the appropriate CH for each one.
**Algorithm 3** Multiple Cluster Algorithm**Input**: *DevicePower, NUMBEROFCLUSTERS* **Output**: *Moving between the Clusters to elect the optimum CH for each* FOR each Cluster i of the network DO     IF (CHcount[i] == NUMBEROFCLUSTERS) THEN   CH [i] = CALL f_AssignClusterHead (i, DevicePower) RETURNING elected CH   prevCH[i] = CH[i]        CHcount[i] = 0     ELSE    CHcount[i]++    IF (prevCH [i]! = 0)       CH[i] = prevCH[i]     END IF END FOR

#### 5.2.4. Data Collection Phase

After selecting the CH, the sink node uses a time programming (TDMA), giving a time limit for each node to transmit its data to the elected CH. In the second stage, the elected CH transmits the received data to the sink sode as scheduled (TDMA). The cluster head, before transmitting the data to the SN, avoids transmitting (Etra) data. The mathematical model and analysis in the coming Section illustrate these ideas precisely. The pseudo-code of Algorithm 4 shows how to determine the current cluster, while the pseudo-code in Algorithm 5 shows how to transmit all cluster data to the sink node (SN).
**Algorithm 4** Identify the cluster of each sensor**Input**: *DeviceId, ClusterElements* **Output**: *Identify the cluster of each sensor* fn_IdentifyCluster (DeviceId) BEGIN For each Cluster i of the network DO     For each sensor j of the Cluster DO          IF (DeviceId == ClusterElements[i][j]) THEN        RETURN i     END FOR END FOR END

**Algorithm 5** Data Transmission Phase**Input**: *ClusterNumber, DevicePower, DeviceId* **Output**: *Transmit all Clusters data to the Sink Node* IF the sensor is the Cluster Head THEN forwards the packets to the Sink Node Else Identify the Cluster and the CH of the current Cluster will be the nextHop ClusterId = fn_IdentifyCluster(DeviceId) nextHop = CH[ClusterId] END IF

**Scheme 2 (Multiple Cluster Algorithm):**

The sensor network is split into different groups; the data is collected from the sensor nodes in each group and transmitted to the corresponding cluster head. In turn, the head of the group sends the collected data to the central management unit sink node. In this case, the energy consumed can be calculated as:

(5) $$E_{CH\text{-}SN=}\sum_{m=1}^{M}\left[\begin{array}{l} \sum_{i}^{N_{m}-1}\left(E_{trans}+\varepsilon amplifcoe\cdot dist_{i}^{{}^{\alpha }}+E_{reciv\text{-}data}\right)\cdot N_{trans}\_dbits\\ +\left(E_{trans}+\varepsilon amplifcoe\cdot dist_{m}^{{}^{\alpha }}\right)\cdot N_{trans}\_dbits_{m} \end{array}\right]$$

where M indicates the cluster’s number, $N_{m}$ indicates the nodes’ number in the corresponding cluster, $dist_{i}$ indicates the distance between the node and its CH, $dist_{m}$ indicates the distance between the CH and the SN, and $N_{trans}\_dbits_{m}$ indicates the number of data bits in the transmitted packet data.

For example, Figure 4 also illustrates the Multiple Cluster Algorithm, which is a process of managing and monitoring the precision agriculture platform, where the network divides into different groups. Each agriculture platform in this scheme is identified as a cluster. The rest of the sensors transmit the obtained data to the corresponding cluster head, then the cluster head transmits the data (service and commands) to the sink node.

## 6. Performance Comparison and Results Analysis

The LEACH protocol is chosen to perform the comparison due to the considerable number of created protocols based on it, such as FBCFP [18] or FL-EEC/D [16].

In this paper, a Lightweight and Efficient Dynamic Cluster Head Election Routing Protocol (LEDCHE-WSN) has been proposed to enhance energy efficiency and extend network life for wireless sensor networks. This algorithm is comprised of two integrated methods: electing the optimum cluster head, and organizing the re-clustering process dynamically. The performance efficiency and precision of the proposed protocol assumptions are validated by using NetSim simulation, and compared with the LEACH and FBCFP protocols. The coming Sections introduce the simulation setup and analysis results.

### 6.1. Simulation Setup

In the simulation setup, 100 sensor nodes are scattered uniformly in a 100 m × 100 m region. The simulation study uses NetSim simulator. The proposed network has been divided into 5 clusters, and each cluster includes 20 nodes. Moreover, each cluster is connected to the corresponding sink node, therefore the number of SNs is also 5, and, in turn, all these SNs transmit their data to the main network station, which is called the “Base Station” (BS). The SN of each cluster is located at the center of the cluster, to aggregate data from all the sensor nodes effectively. Therefore, all nodes of the cluster can be connected with it using the single-hop model method. Besides that, sink nodes are distributed to make the network more scalable. Additionally, the BS is located at the center of each network. The BSs collect their data from rechargeable SNs. To be more specific, in the proposed protocol there are 5 SNs in a 100 × 100 m network size, which means there is one SN in each 20 m × m, each SN is at the center of their area, meaning the distance between the regular nodes and the SN is only approximately 10 m. Therefore, the nodes are not long-distance from the SN. Moreover, the BS is at the center of the 5 SNs, in the same way; the distances become closer. Hence, good architecture designing makes the connectivity more simple and flexible. Table 2 shows the network parameters and corresponding values.

### 6.2. Results Analysis

The proposed LEDCHE-WSN protocol is evaluated and compared with the LEACH and FBCFP protocols in terms of energy consumption, mean package delay, and total packets dropped (loss ratio). Additionally, it is compared with existing HEED and FLCFP performance in terms of network lifetime.

The proposed agorithm is characterized by simplicity, ease, and lightness as it was pointed in the paper title. The computational complexity burden was avoided in the proposed algorithm compared with other protocols. Most of the previous studies, including the FBCFP and LEACH protocols, adopted a heavy measuring method in choosing the optimum cluster head, such as measuring the signal strength, residual energy, node density, hop count, and the distance among nodes and base station which causes delays, increasing loss ratio, and draining the network energy that is supposed to be provided. More precisely, the FBCFP protocol makes extensive calculations in the cluster head election process, such as node density, CH degree count, traffic level, and bandwidth availability. On the other hand, the LEACH protocol calculates and compares the remaining energy for a CH candidate with all other sensor nodes, causing shortening of the network lifetime and adding more burdens to the network. On the other hand, the LEDCHE-WSN algorithm checks the remaining energy only to ensure that a CH candidate is alive, without adding a burden in calculation and comparison for the remaining energy of the CH candidate with all the other sensor nodes. As a result, the experiments in the coming sections prove the simplicity and lightness of the proposed algorithm compared to the most recent studies.

#### 6.2.1. Energy Consumption and Network Lifetime

The total energy consumption measures the energy consumed by each node in forwarding packets to sink nodes, which indicates the network lifetime wasted by the protocols. The calculation of Average Energy Consumption uses Equation (6).

(6) $$\mathrm{Average}\;\mathrm{Energy}\;\mathrm{Consumption}\left[j\right]=\frac{\mathrm{Total}\;\mathrm{Energy}\;\mathrm{consumption}}{\mathrm{Total}\;\mathrm{Number}\;\mathrm{of}\;\mathrm{received}\;\mathrm{packets}}$$

**Figure 7:**
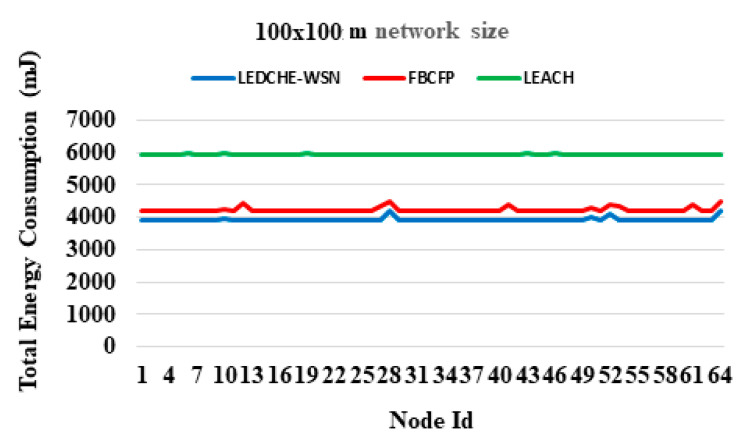
Total energy consumption for each node considering a 100 × 100 m network size for LEACH, FBCFP, and LEDCHE-WSN protocols.

Figure 7 plots the total energy consumption per node, including the nodes ID. As may be seen, it can be noted that the proposed LEDCHE-WSN protocol reduced the total energy consumption of the sensor nodes by approximately 32% when compared to the LEACH protocol, and 8% when compared to the FBCFP protocol for the 100 × 100 m network size. This can occur for several reasons. First, it can occur due to deleting failed or dead nodes from the network topology during the re-clustering process to address the black holes and routing delay problems, which was resulting in a waste of time and energy due to additional control messages for useless nodes, if they are not deleted from the topology list. Second, it can happen due to relieving regular nodes from the processes of selecting the next CH node. Instead, it was assigned all the previous functions to the rechargeable SN nodes, contrary to what happened in the previous studies, especially in the FBCFP protocol. Finally, the reduced energy consumption could be due to the fact that, checking the remaining energy in LEDCHE-WSN is performed only to ensure that, the CH candidate is alive. Whereas the FBCFP and LEACH protocols calculate and compare the remaining energy for a CH candidate with all other sensor nodes, causing in shortening the network lifetime and adding more burdens to the network. This figure presents the total energy consumption for each node when the last node of the LEACH protocol are dead, and the simulation time is finished (1200 s is the simulation time, as shown in Table 2). A good load balance is estimated inside the proposed protocol by monitoring the total energy consumption for each node. From Figure 7, it is observed that the total energy consumption in the proposed protocol, until the simulation time is finished, is much more regular and the energy is distributed more equally among network sensors than total energy consumption for the benchmarks, which means energy distribution in the proposed protocol is better, therefore, proves the efficiency of the proposed protocol.

According to [18], considering results for 100 nodes, the HEED protocol improves the LEACH protocol network lifetime by 8%, the FLCFP protocol improves the LEACH by 10%, and the FBCFP protocol improves the LEACH by 25%. This means that the proposed protocol LEDCHE-WSN overcomes the performances of LEACH, HEED, FLCFP, and FBCFP in terms of network lifetime.

#### 6.2.2. Mean Package Delay

Mean delay $\left(Mean_{dly}\right)$ is the sum of Queuing Delay ($Que_{dly}$), Total Transmission Time $\left(Total_{transtime}\right)$, and Routing Delay $\left(Rout_{dly}\right)$. Mean Package delay can be calculated by Equation (7).

(7) $$Mean_{dly}=Que_{dly}+Total_{transtime}+Rout_{dly}$$

The Total expected Queuing Time is as in Equation (8):

(8) $$Queuing\begin{array}{cc} Time & = \end{array}T=\frac{1}{2\mu }\times \frac{\rho }{1-\rho }$$

where: μ= service rate, i.e., the time is taken to service each packet.

ρ is the utilization given as follows, $\rho =\frac{\lambda }{\mu }$

λ= Arrival time, i.e., the rate at which packets arrive.

Total Transmission Time is the sum of transmission time through each link.

Transmission time through each link is the same as presented in Equation (9):

(9) $$Transmission\;time\;through\;each\;link=\frac{Payload\begin{array}{cc} size & \left(Bytes\right) \end{array}\times 8}{Uplink\begin{array}{cc} speed & \left(bps\right) \end{array}}$$

where the payload size is the sum of packet size and the overhead as presented in Equation (10):

(10) $$Payload\begin{array}{cc} size & =Packet\;Size+Overhead \end{array}$$

Routing Delay is approximately 1 micro sec, and can be calculated from the event trace file. It is the difference between “Physical In” and “Physical Out” time.

**Figure 8:**
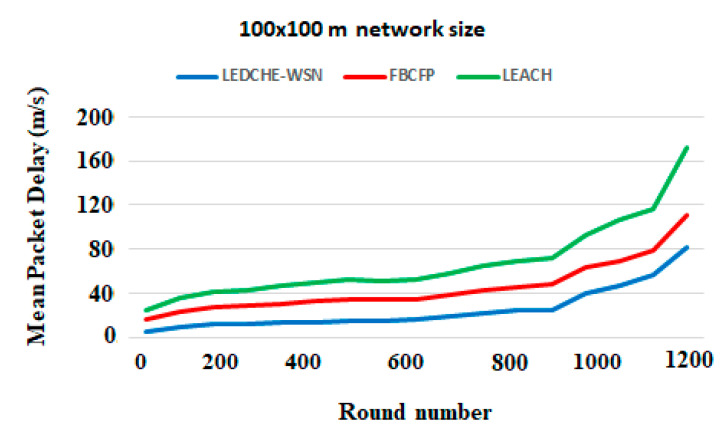
Mean package delay considering 100 × 100 m network size for LEACH, FBCFP, and LEDCHE-WSN protocols.

Figure 8 plots the $\left(Mean_{dly}\right)$ considering the round’s number. From Figure 8, it can be observed that the proposed protocol improves $\left(Mean_{dly}\right)$ by more than 42% compared to the LEACH protocol, and more than 15% compared to the FBCFP protocol; this is a consequence of using the single-hop clustering model at cluster core, resulting in the fast delivery process and making it much more simple and lightweight. The LEACH and FBCFP protocols adopted a multi-hop model and synchronization of multi-hop time in the cluster core, producing a very high load and data transmission among nodes, causing much more packet delay and packet loss, and therefore quick energy depletion. Similarly, using the random method in the LEACH protocol during the CH election process causes much more delay, due to the false election of the failure nodes (black holes) and much more time needed to get out of those holes. Additionally, the extensive calculation in the cluster head election process, such as node density, CH degree count, traffic level, and bandwidth availability in the FBCFP protocol results in more delays and increased complexity, as detailed in Section 6.2. (Results Analysis). On the other hand, the LEDCHE-WSN protocol checks nodes are alive before the election process to overcome the black holes problem. Furthermore, by locating the sink node at the center of the cluster, the distance between nodes and sink nodes is reduced. Consequently, mean package delay is improved, and data aggregation from all sensor nodes is performed effectively.

#### 6.2.3. Total Packets Dropped (Loss Ratio)

Considering packet loss in the selection of the best communication path has a significant impact on reducing energy consumption and increasing the network throughput. Moreover, it is used as a measure to determine the efficiency of routing protocols. The loss ratio can be calculated using Equation (11).

(11) TPD=Total Packets Sent−Total Packets Received

where TPD indicates, the total packets dropped during the transmission process.

**Figure 9:**
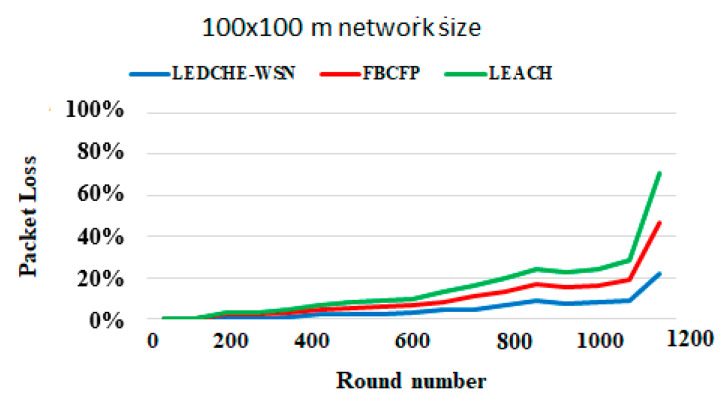
Percentage of packets loss ratio for the 100 × 100 m network size for LEACH, FBCFP, and LEDCHE-WSN protocols.

Figure 9 shows the total packets dropped (loss ratio) considering the round’s number. As may be observed, the proposed LEDCHE-WSN protocol reduced the loss ratio by more than 46% compared to the LEACH protocol, and more than 25% compared to the FBCFP protocol. This is due to adopting the single-hop clustering model that is easy to implement and fast to deliver, but its main drawback appears when the network size grows. To overcome this problem, LEDCHE-WSN architecture is enhanced by using a single-hop model at the cluster core where it nodes are a small size have a short distance between one another. Moreover, using a multiple-hop model at the cluster core causes multiple congestion points in the cluster, which in turn results in quick node energy depletion. Consequently, the core becomes lightweight and the loss ratio is improved. The LEACH FBCFP protocols adopted a multiple-hop model at cluster core, producing a very high load and data transmission among nodes, causing more packet delay, packet loss and, therefore, quick energy depletion. Furthermore, repetitive checking at each round/cycle for dead nodes, in the LEACH and FBCFP protocols, caused more delays and increased the loss ratio. The proposed LEDCHE-WSN protocol is distinguished by deleting the failure and/or dead nodes from the network topology during the re-clustering process to address the black holes and routing delay problems.

### 6.3. Proposed Protocol Strength Points Compared with LEACH and FBCFP Protocols

The proposed LEDCHE-WSN protocol is preferable to the benchmark LEACH and FBCFP protocols, taking into account the following aspects:

- The LEDCHE-WSN protocol adopted a hybrid clustering model. A single-hop model is used at the cluster core, where the nodes are a small size and have a short distance between one another, making it more simple, fast, lightweight, and easy to deploy. On the other hand, a multi-hop model is used at the cluster edge to make it more connective and scalable. LEACH and FBCFP protocols use the multi-hop model at the cluster core. Despite the fact a multi-hop model can improve connectivity and extend network coverage, it causes more packet delay and packet loss, especially at the cluster core. As is well-known, the direct routing approach (single-hop model) is easy to deploy and provides fast delivery, but its drawback appears when the network size grows. Therefore, this problem was overcome by improving the LEDCHE-WSN architecture through distributing sink nodes, and using the multi-hop model at the edge of the cluster to make it much more connective and scalable;
- The LEACH protocol elects the cluster-head randomly, which results in an unbalanced energy level and thus dissipates the total network energy. The proposed algorithm performs a periodical election by excluding failed or dead nodes, which leads to a balanced energy level;
- The proposed algorithm works randomly only at the beginning of each round, to enhance the privacy of location, minimize hackers’ chances of successive packets, and further complicate guessing the next packet;
- The random method in the LEACH protocol caused much more delay, due to the false election of the failure nodes (black holes) and much more time needed to get out of those holes. On the other hand, the proposed protocol ensures the living of the node by checking the remaining power during the random election for the CH candidate to overcome the black holes problem, and that happened only at the beginning of each round;
- The LEACH protocol calculates the remaining energy and times of being elected as a cluster head for each sensor node adds more burden to the network. Moreover, the FBCFP protocol is more complicated, focusing on the degree of accuracy to choose the cluster head performing complex mathematical operations and measuring multiple factors (such as node density, CH degree count, traffic level, and bandwidth availability), causing more delays and increasing complexity. The LEDCHE-WSN algorithm checks the remaining energy just to ensure that a CH candidate is alive, without adding a burden in calculation and comparison for the remaining energy of the CH candidate with all the other sensor nodes as detailed in Section 6.2. (Results Analysis);
- More importantly, the LEDCHE-WSN protocol relieved regular sensors from the processes of selecting the next CH node and notifying the rest of the cluster nodes accordingly. Instead, it assigned all previous jobs to the rechargeable SN nodes, contrary to what happened in most of the previous studies—including the FBCFP protocol. In this protocol, cluster nodes participate in selecting the next CH node and, then, notifies the rest of the cluster nodes. It keeps all the information of the topology list, leading to a significant and effective drain on cluster nodes’ energy;
- The proposed method is distinguished from the LEACH and FBCFP protocols by addressing the problem of re-electing a failed or dead node, checking the status of the node, and then deleting the failed and/or dead nodes from the network topology list, resulting in extending the overall network lifetime.

## 7. Conclusions and Future Works

Wireless sensor networks have limited resources in terms of bandwidth, processing, and storage capabilities. Clustering technologies execute an important function in energy efficiency, network extension, and overhead reduction, resulting in much more efficient WSNs. Cluster head election is a major challenge for enhancing energy efficiency, and spans a network lifetime. In this paper, the LEDCHE-WSN protocol has been proposed to enhance energy efficiency and extend the network life for wireless sensor networks. The proposed algorithm’s mathematical modeling and analysis are introduced to illustrate the main idea much more. The proposed protocol improvements were displayed in comparison with the benchmarks LEACH protocol. Extensive experimental results revealed the proposed protocol outperforms the LEACH protocol by approximately 32%, and the FBCFP protocol by 8% in terms of power consumption and network lifetime. Concerning mean package delay, LEDCHE-WSN improves the LEACH protocol by 42%, and the FBCFP protocol by 15%. In terms of loss ratio, it improves the LEACH protocol by 46%, and the FBCFP protocol by 25%. Similarly, the HEED protocol improves the LEACH protocol network lifetime by 8%, and the FLCFP protocol improves the LEACH by 10%. This means that the proposed protocol, LEDCHE-WSN, overtakes the performances of LEACH, HEED, FLCFP, and FBCFP in terms of network lifetime.

Future trends of this research study may include how to face the challenge of elected CH node failure while performing its work. Further, a scenario of scattering a considerable number of sensor nodes randomly and evaluating the effect of packet loss in the routing process, using the obtained measurements. Moreover, experiments should be performed to determine the optimal number of sink nodes and the optimal number of regular nodes in each cluster. Furthermore, for future work, it is necessary to evaluate this work using the metrics of the first death, and half of the live nodes. The challenges of routing and data transmission between the sink nodes and the corresponding BS is also left for future work. Ultimately, the mobility of sensor nodes will be considered, and extend this work to the Internet of Things world.

## Figures and Tables

**Table 1:** Summary of related work for clustering protocols.

Protocol	Year	Technique	Classification	Strengths	Weaknesses
Proposed protocol (LEDCHE-WSN)	2021	Combined CH election Method Self-configuring cluster formation	Hybrid clustering scheme	(1) Introduces a new and efficient method to elect the CH; (2) Simple, easy, and light; (3) Deletes the dead nodes from the topology list; (4) Self-configuring the re-clustering process dynamically; (5) Complexes tracking the CHs by attackers	Ignore to face the challenge of electing CH node dead/failure while performing its work (Future work)
FBCFP protocol [18]	2019	Neuro-fuzzy rule	Adaptive schemes	It uses a deep learning-based method to ensure electing the optimum CH	Extensive calculation in the cluster head election process drains the cluster nodes’ energy quickly and adds more burdens to the network.
Energy Optimization in Cluster-Based Routing Protocols for Large-Area Wireless Sensor Networks [24]	2019	Derivative-free Nelder–Mead Simplex method	Hybrid clustering scheme	The proposed protocol decreases energy consumption effectively	The heavy calculations of the transmission range of the control messages and the average number of clusters in each round result in a great delay of operations, and the depletion of the network’s energy
DFLCHES Scheme [25]	2018	Genetic algorithms /Elbow method/Fuzzy predictive method	Adaptive schemes	(1) Effective cluster head selection that enhancement the lifetime of the network; (2) Out-performed the KBPSO and LEACH schemes in terms of packet drop ratio, total energy consumption, and number of average delays.	The extensive calculation, such as node centrality measure, hop count, and density, resulting in quick energy depletion, consequently shortening the lifetime of the network
A TOPSIS Based Cluster Head Selection for Wireless Sensor Networks [5]	2016	TOPSIS algorithm and measures the (distance to BS, transmission rate, Residual energy)	Combined metric schemes	Improve the lifetime of the network compared to LEACH and AHP	Focusing on the accuracy degree at the expense of the new burdens that can be added in terms of mathematical complexity and increasing of signals
Clustering Approach in Wireless Sensor Networks Based on K-means: limitations and Recommendations [26]	2015	Residual energy measuring method and the nodes’ threshold value	Adaptive schemes	Better nodes working in the network in comparison to the LEACH	Consumed power in the residual energy measuring and calculated it with the threshold value for all nodes, itself considered as an additional burden on the network
Energy Efficient distributed cluster head scheduling scheme for two-tiered WSNs [25]	2015	Remaining energy/Cluster optimal centrality degree	Adaptive schemes	Overcome frequent election of cluster head and fair energy load balancing among cluster-based networks	The residual energy level measurement and signal strength to each node adds more burdens to the network, resulting in more energy consumed
DCPVC Protocol [27]	2015	Calculating the remaining energy and the times of being elected as cluster head	Combined metric schemes	Energy consumption and better construction time	Calculating the remaining energy and election times as the cluster head for each sensor node adds more load to the network.
Cluster head selection optimization based on genetic algorithm [28]	2015	Calculating the residual energy, inter-and intra-cluster distance	Adaptive scheme	Good lifetime and network load balancing	Repetitive residual energy and distances calculation adds more burdens, leading to whole-network delay and quick power depletion
DCHEP [29]	2015	Remaining energy measuring and other sensors connectivity	Hybrid clustering scheme	(1) Scalable and flexible solution targeting intense WSNs with random mobility; (2) Achieves high growth in energy performance and availability compared to LEACH.	(1) Residual energy calculated for the nodes is unsuccessful in the case of the nodes’ rapid movement; (2) Resultsin continuous connectivity loss.
The Energy Efficient Multi-Hop Clustering Process for Data Transmission in Mobile Sensor Networks [30]	2014	Energy level Measuring, Stability, Connectivity	Hybrid clustering scheme	(1) Introduces high bandwidth and low delay in the network; (2) Periodical cluster head election is enhanced to form effective routing and reduce energy consumption; (3) Good connectivity and stability.	(1) Periodically maintaining the routing table makes the security side weak; (2) Residual energy calculating of the nodes is often unsuccessful in rapid nodes movement, especially in the critical cases; (3) Delays due to the failure of some nodes.
LEACH [31]	2002	A random CH election Adaptive, self-configuring cluster formation	Deterministic schemes	(1) Distributes power waste evenly across the sensors; (2) High scalability; (3) Robusts the security side; (4) Simple, low overhead, and fast.	(1) The random method adds more limitations; (2) Some nodes cause a waste of energy in transmitting data to an area too far from cluster heads; (3) High redundancy of data.

**Table 2:** Network Simulation Parameters.

Value	Parameter
100 × 100 m	Network size
Sensor	Device Type
100	Number of sensor nodes
1460	MTU (bytes)
DSR	WSNs Routing Protocols
PHY IEEE802.15.4	MAC Layer Protocol
512 bits	Control packet size
4000 bits	Data packet size
6 J = 6000 m J	Initial Energy
50 nj/bit	Eelect
CARRIER_SENSE_ONLY	CCA
−95	Packet Reception Power Threshold (dBm)
−85	Receiver Sensitivity (dBm)
100	Sensor Range (m)
250	Data Rate (Kbps)
1200	Simulation Time (s)
NetSim	Simulator

## References

[1] Sarkar A., Murugan T.S. (2019). Cluster head selection for energy efficient and delay-less routing in wireless sensor network. Wirel. Netw..

[2] Shankar T., Shanmugavel S. (2014). Energy Optimization in Cluster based Wireless Sensor Networks. J. Eng. Sci. Technol..

[3] Gherbi C., Zibouda A., Mohamed B. (2019). A Novel Load Balancing Scheduling Algorithm for Wireless Sensor Networks. J. Netw. Syst. Manag..

[4] Wang J., Gao Y., Liu W., Sangaiah A.K., Kim H. (2019). An improved routing schema with special clustering using PSO algorithm for heterogeneous wireless sensor network. Sensors.

[5] Al-Baz A., El-Sayed A. (2018). A new algorithm for cluster head selection in LEACH protocol for wireless sensor networks. Int. J. Commun. Syst..

[6] Jan S.R.U., Jan M.A., Khan R., Ullah H., Alam M., Usman M. (2019). An energy-efficient and congestion control data-driven approach for cluster-based sensor network. Mob. Netw. Appl..

[7] Roopali P., Rakesh K. (2019). Technological aspects of WBANs for health monitoring: A comprehensive review. Wirel. Netw..

[8] Khedr A.M., Osamy W., Salim A. (2018). Distributed coverage hole detection and recovery scheme for heterogeneous wireless sensor networks. Comput. Commun..

[9] Farman H., Javed H., Jan B., Ahmad J., Ali S., Khalil F.N., Khan M. (2017). Analytical network process based optimum cluster head selection in wireless sensor network. PLoS ONE.

[10] El-Refaay S., Azer M.A., Abdelbaki N. Cluster Head Election in Wireless Sensor Networks. Proceedings of the 10th International Conference on Information Assurance and Security.

[11] Qu Y., Zheng G., Ma H., Wang X., Ji B., Wu H. (2019). A survey of routing protocols in WBAN for healthcare applications. Sensors.

[12] Zhansheng C., Hong S. (2018). A grid-based reliable multi-hop routing protocol for energy-efficient wireless sensor networks. Int. J. Distrib. Sens. Netw..

[13] Ruisong H., Wei Y., Yipeng W., Kaiming Y. (2017). DCE: A distributed energy-efficient clustering protocol for wireless sensor network based on double-phase cluster-head election. Sensors.

[14] Elhoseny M., Aboul Ella H. (2019). Hierarchical and clustering WSN models: Their requirements for complex applications. Dynamic Wireless Sensor Networks.

[15] Heinzelman W.R., Anantha C., Hari B. Energy-efficient communication protocol for wireless microsensor networks. Proceedings of the 33rd annual Hawaii International Conference on System Sciences.

[16] Hamzah A., Shurman M., Al-Jarrah O., Taqieddin E. (2019). Energy-Efficient Fuzzy-Logic-Based Clustering Technique for Hierarchical Routing Protocols in Wireless Sensor Networks. Sensors.

[17] Sinde R., Begum F., Njau K., Kaijage S. (2020). Refining Network Lifetime of Wireless Sensor Network Using Energy-Efficient Clustering and DRL-Based Sleep Scheduling. Sensors.

[18] Thangaramya K., Kulothungan K., Logambigai R., Selvi M., Sannasi G., Kannan A. (2019). Energy aware cluster and neuro-fuzzy based routing algorithm for wireless sensor networks in IoT. Comput. Netw..

[19] Kumar R., Logeswari R., Devi N., Bharathy S. (2017). Efficient clustering using ECATCH algorithm to extend network lifetime in wireless sensor networks. Int. J. Eng. Trends Technol..

[20] Ding X., Sun X., Huang C., Wu X. (2016). Cluster-level based link redundancy with network coding in duty cycled relay wireless sensor networks. Comput. Netw..

[21] Rana S., Bahar A., Islam N., Islam J. (2015). Fuzzy based energy efficient multiple cluster head selection routing protocol for wireless sensor networks. Int. J. Comput. Netw. Inf. Secur..

[22] Kang S.H. (2019). Energy Optimization in Cluster-Based Routing Protocols for Large-Area Wireless Sensor Networks. Symmetry.

[23] Zebbane B., Chenait M., Benzaid C., Badache N. (2018). RTCP: A redundancy aware topology control protocol for wireless sensor networks. Int. J. Inf. Commun. Technol..

[24] Ramakrishnan S., Shyry S.P. (2018). Distributed fuzzy logic based cluster head election scheme (DFLCHES) for prolonging the lifetime of the wireless sensor network. Int. J. Eng. Technol..

[25] Hassan A.A.H., Shah W., Husein A.M., Talib M.S., Mohammed A.A.J., Iskandar M. (2019). Clustering approach in wireless sensor networks based on k-means: Limitations and recommendations. Int. J. Eng. Trends Technol..

[26] Hamzeloei F., Dermany M.K. (2016). A TOPSIS based cluster head selection for wireless sensor network. Procedia Comput. Sci..

[27] Kannan G., Sree R.R.T. (2015). Energy efficient distributed cluster head scheduling scheme for two tiered wireless sensor network. Egypt. Inform. J..

[28] Hematkhah H., Yousef S.K. (2015). DCPVP: Distributed clustering protocol using voting and priority for wireless sensor networks. Sensors.

[29] Pal V., Girdhari S., Yadav R.P. (2015). Cluster head selection optimization based on genetic algorithm to prolong lifetime of wireless sensor networks. Procedia Comput. Sci..

[30] Yahya H., Yaarob A., Kemp A.H. (2015). A dynamic cluster head election protocol for mobile wireless sensor networks. Proceedings of the 2015 International Symposium on Wireless Communication Systems (ISWCS).

[31] Sundaram V.S. (2014). The Energy Efficient Multi-Hop Clustering Process for Data Transmission in Mobile Sensor Networks. Int. J. Comput. Sci. Mob. Comput..

[32] Mudathir F.S.Y., Rodrigues J.J.P.C., Khalifa O.O., Mohammed A.B., Korotaev V. (2020). Service Redundancy and Cluster-based Routing Protocols for Wireless Sensor and Mobile Ad-Hoc Networks: A Survey. Int. J. Commun. Syst..

[33] Li D.A., Hao H., Ji G., Zhao J. (2015). An adaptive clustering algorithm based on improved particle swarm optimisation in wireless sensor networks. Int. J. High Perform. Comput. Netw..

[34] Behera T.M., Mohapatra S.K., Samal U.C., Khan M.S., Daneshmand M., Gandomi A.H. (2019). Residual Energy-Based Cluster-Head Selection in WSNs for IoT Application. IEEE Internet Things J..

[35] Nokhanji N., Hanapi Z.M., Subramaniam S., Mohamed M.A. (2015). An energy aware distributed clustering algorithm using fuzzy logic for wireless sensor networks with non-uniform node distribution. Wirel. Pers. Commun..

[36] Nawaz Jadoon R., Zhou W., Khan I.A., Khan M.A., Jadoon W. (2019). EEHRT: Energy efficient technique for handling redundant traffic in zone-based routing for wireless sensor networks. Wirel. Commun. Mob. Comput..

